# Determination of Capillary Blood TSH and Free Thyroxine Levels Using Digital Immunoassay

**DOI:** 10.1210/jendso/bvae030

**Published:** 2024-02-14

**Authors:** Nami Suzuki, Miki Takeuchi, Naoko Miyazaki, Katsumasa Tanaka, Sosuke Utsunomiya, Yoshiyuki Arai, Toru Yoshimura, Kiminori Sugino, Koichi Ito, Jaeduk Yoshimura Noh

**Affiliations:** Department of Internal Medicine, Ito Hospital, Tokyo 150-8308, Japan; Diagnostic Division, Abbott Japan LLC, Matsudo, Chiba 270-2214, Japan; Clinical laboratory, Ito Hospital, Tokyo 150-8308, Japan; Clinical laboratory, Ito Hospital, Tokyo 150-8308, Japan; Diagnostic Division, Abbott Japan LLC, Matsudo, Chiba 270-2214, Japan; Diagnostic Division, Abbott Japan LLC, Matsudo, Chiba 270-2214, Japan; Diagnostic Division, Abbott Japan LLC, Matsudo, Chiba 270-2214, Japan; Department of Surgery, Ito Hospital, Tokyo 150-8308, Japan; Department of Surgery, Ito Hospital, Tokyo 150-8308, Japan; Department of Internal Medicine, Ito Hospital, Tokyo 150-8308, Japan

**Keywords:** capillary blood, thyroid stimulating hormone, TSH, free thyroxine, digital immunoassay

## Abstract

**Background:**

The remote performance of thyroid function blood tests is complicated because it requires blood collection.

**Objective:**

To compare TSH and free thyroxine (FT4) levels between capillary and venous blood and assess the adequacy of measuring each value in capillary blood.

**Methods:**

This prospective intervention study was conducted at Ito Hospital and was based on the clinical research method. The participants were 5 healthy female volunteers and 50 patients (41 females and 9 males) between the ages of 23 and 81 years. To measure TSH and FT4 levels in capillary and venous blood, a digital immunoassay (d-IA) method capable of measuring trace samples was used. Chemiluminescence measurements were used as controls. Values obtained for each assay system were compared using Spearman's correlation analysis. Capillary blood was collected using an autologous device (TAP II; not approved in Japan).

**Results:**

Capillary plasma volume obtained using TAP II was 125 µL or more in 26 cases, 25 µL to 124 µL in 24 cases, and less than 25 µL in 5 cases. Strong correlations were noted in the TSH and FT4 levels between capillary and venous blood, with correlation coefficients of rs = 0.99 and rs = 0.97, respectively.

**Conclusion:**

Capillary TSH and FT4 levels strongly correlate with venous blood values. Trace samples can be used in high-precision d-IA methods. These results may promote telemedicine in assessing thyroid function.

The COVID-19 pandemic reduced face-to-face patient visits [1] and has accelerated the need for telemedicine. In some fields where blood testing is not required, telemedicine could be an alternative if telemedicine devices are available. However, for thyroid diseases, especially thyroid dysfunction, blood testing to measure thyroid hormone levels is essential for accurate assessment. Self-sampling of capillary blood using a microneedle device is one way of self-collection of blood [2, 3], and this method has already been used in patients with diabetes to check glucose levels [4]. Although the currently used fingertip pricking with microneedle method is accepted worldwide and is less painful than venous blood sampling, this method can collect only 3 µL of capillary blood [5]. Conversely, a microneedle capillary blood collection device enabled us to collect 100 μL of capillary blood samples from the upper arm [6] with less pain than usual venous blood sampling or fingertip pricking method [3, 5]. Some studies that used this device have revealed that major immune cells [7]; SARS-CoV-2 antibody [8]; and biochemical components including liver enzymes [9], autoantibodies [10, 11], and inflammatory markers [10-12] can be measured as accurately as in venous blood samples. Capillary blood can be collected using a microneedle sampling device; however, its volume is still insufficient for blood testing using conventional instruments. The recently developed ultra-high-sensitivity digital immunoassay (d-IA) with attomolar sensitivity enables the detection of a single enzyme using single-molecule detection technology [13]. The d-IA can detect enzymes in a small amount of samples. The d-IA has substantial potential, and it has already been used in the medical field [14]. The d-IA technique was used in this study to measure a small amount of specimens.

Regarding thyroid function, capillary blood sampling from heel prick has been used for screening congenital hypothyroidism of newborns worldwide [15-17], and this method has been implemented as a public service since 1977 in Japan. This screening test has revolutionized the detection of congenital metabolic and endocrine diseases because treatments can be initiated promptly in the early stages of life. Moreover, according to the American Thyroid Association statement formulated in 2018 [18], although point-of-care (POC) thyroid diagnostic kits are used worldwide, most of these are qualitative TSH measuring kits designed for screening congenital primary hypothyroidism. Additionally, the few POC quantitative assay kits for TSH, T4, and T3 are not yet commercially available in most countries. A recent study that analyzed thyroid hormone data obtained from a commercially available testing kit using capillary blood collected by the finger prick method revealed the prevalence of potential thyroid dysfunction among the general population [19]. However, the study did not evaluate the accuracy of the test results. The limitations of POC diagnostic test kits include a lack of sensitivity and accuracy, the ability to measure items only in narrow ranges, and unreliability of results when using whole blood as a test sample [18].

Kahaly et al [20] and Shurbaji et al [21] observed significant correlations and concordance in the levels of TSH between capillary blood, venous whole blood, and venous serum using a Wondfo commercially available testing kit and compared the values to Abbott and Roche conventional kits as reference methods. To our knowledge, no previous study has assessed the concordance of both TSH and free T4 (FT4) thyroid hormone levels simultaneously between capillary and venous blood samples. The primary purpose of this study was to compare the values of TSH and FT4 levels in the capillary blood with those in the venous blood to assess adequacy and concordance.

## Materials and Methods

### Study Design and Participants

**Figure 1.**
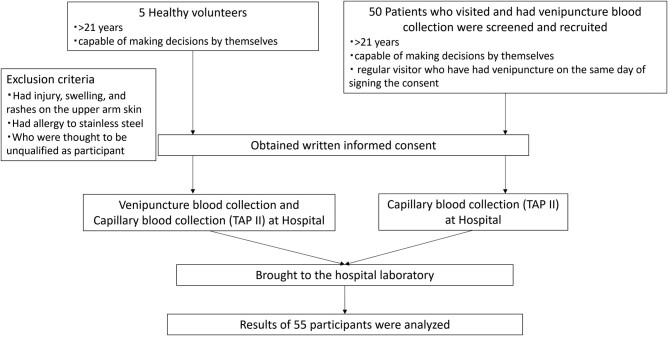
Participants’ flow charts. The participants consisted of 5 healthy volunteers and 50 patients. The inclusion criteria for the volunteers were (1) healthy male or female aged over 21 years and (2) capable of making decisions and for the patients were (1) aged over 21 years, (2) regular visitors who underwent venous blood collection on the same day of signing the consent form, and (3) capable of making decisions. The exclusion criteria for all participants were as follows: (1) injury, swelling, and rashes on the upper arm skin, where blood collection was performed; (2) allergy to stainless steel because the micro-needle brads are made of stainless steel; and (3) those who were thought to be unqualified as participants.

Based on the clinical research method, this study was conducted as a prospective, single-center, intervention study of a microneedle self-blood collection equipment at Ito Hospital between July 2022 and October 2022. The protocol was submitted to the Japanese Ministry of Health, Labour, and Welfare and approved by the Clinical Research Review Board of the University of Tokyo (#CRB3180024) and the Ethics Committee of Ito Hospital (#IRB364). The participants were 5 healthy female volunteers recruited by a volunteer recruitment agency (NEW-ING NPO Corp.) and 50 patients observed at Ito Hospital with various thyroid functions. All participants met the inclusion and exclusion criteria described in the registered study protocol and provided written informed consent to participate (Fig. 1).

The primary objective was to assess the concordance of TSH and FT4 values with capillary blood acquired via TAP II (YourBio Health, Medford, MA, USA; not approved in Japan) compared with venipuncture samples. The secondary objective was to measure the values of biochemical parameters, including aspartate aminotransferase (AST), alanine aminotransferase (ALT), total bilirubin (T-Bil), and creatinine (CRE), in capillary blood and to assess the concordance with those values in venous blood.

### Blood Collection

Venous blood sampling was performed by clinical laboratory technologists using the standard venipuncture method. Venous blood samples were placed at room temperature for 10 to 15 minutes until clotting was complete and then centrifuged for 7 minutes at 3000 rpm.

Capillary blood samples were collected from the upper arm using an autologous microneedle blood collection device, TAP II. Capillary blood collection was assisted by 3 designated nurses (M.A., S.M., N.I.) using a TAP II device and a tube containing lithium heparin anticoagulant, following the instructions for using it provided by the company.

### Laboratory Methods

To measure TSH and FT4 values, a d-IA method [14] capable of measuring trace samples was used for capillary and venous blood measurements. The d-IA reagent for TSH and FT4 was developed by Abbott Japan LLC. TSH and FT4 assays in d-IA were performed in a two-step reaction using paramagnetic microparticle enzyme immunoassay technology with fluorogenic substrates. The coefficients of variants (CV) of the d-IA TSH assay were less than 10% [22]. Five microliters for TSH or 9 μL for the FT4 assay, specific antibody-coated paramagnetic microparticles, and alkaline phosphatase-labeled conjugates were combined to form beads with antigen-antibody complexes. The resulting bead and fluorescent images were captured using a custom-made camera. Image analysis was described as reported [14, 22]. To measure TSH and FT4 using d-IA, the same sample was repeatedly used according to the amount of sample collected. TSH was measured twice at maximum with the same sample, and FT4 was measured 3 times at maximum, and then the mean value was calculated. Conventional chemiluminescent measurement kits [ARCHITECT TSH (AB_2883972) and FT4 (AB_2801665), Abbott Laboratories] were used as a control. According to the package inserts, the CV of the Architect TSH and FT4 assay are both designed to have a precision of <10% (total CV). Biochemical parameters, including AST (Sekisui Medical Co., Ltd.), ALT (Sekisui Medical Co., Ltd.), T-Bil (FUJIFILM), and CRE (Kainos), were measured using an automatic biochemical analyzer (BioMajesty® JCA-ZS050, JEOL Ltd.), and a sample cup for a small number of specimens.

### Statistical Analysis

Data were expressed as mean and as median and range for age. Spearman's correlation analysis was used to compare the values obtained in each assay system, and Passing–Bablok regression analysis was used to calculate the slope of the regression line. Statistical analyses were conducted using R version 4.2, R studio 2022.2.2.485, and R Package mcr version 1.2.2 (R Foundation for Statistical Computing, Vienna, Austria). The statistical significance was set at a *P*-value of <.05.

## Results

### Participant Demographics

Fifty-five participants were enrolled in the study: 5 healthy female volunteers and 50 patients with various thyroid functions (9 males and 41 females). The median age was 52 years (range: 23-81 years), and the detailed thyroid conditions of the participants are shown in Table 1. Of the 50 patients, 41 were on medication, and 4 of the 50 patients were untreated. Four of the 41 patients on medication had undergone curative treatment, and 1 relapsed after curative treatment. Although we provided equal opportunities for male and female patients to participate in this study, most male patients refused to be enrolled.

**Table 1.** Participant demographics

Sex	F:M = 46:9	
Age: median (range)	52 years (23-81)	
Thyroid condition	Graves’ disease	30
	Hashimoto's disease	13
	Others	7
	Hypothyroidism	3
	Painless thyroiditis	2
	Thyroid nodules	1
	SITSH	1
	Healthy volunteers	5
Treatment	On medication	41
	Post-curative treatment	4
	Relapsed after treatment	1
	Untreated	9

Abbreviation: SITSH, syndrome of inappropriate secretion of TSH.

### Blood Collection

Capillary plasma volume obtained using TAP II was 125 µL or more in 26 cases, 25 μL to 124 µL in 24 cases, and less than 25 µL in 5 cases. Our protocol allowed us to use TAP II only once per participant, so even though we could not obtain enough capillary blood, we could not repeat the second TAP II use. The 50 participants whose capillary TAP II blood volume exceeded 25 µL were assigned to the full-analysis set (FAS) group, and 24 of the 50 participants whose TAP II blood volume was not enough to measure biochemical items were assigned to the per-protocol set (PPS) group. Five participants whose capillary blood volume was less than 25 μL could not perform the measurement process. No adverse events related to the use of TAP II were reported after the procedure.

### Analyses of TSH and FT4 Levels Between Capillary (TAP II) and Venous Blood

**Figure 2.**
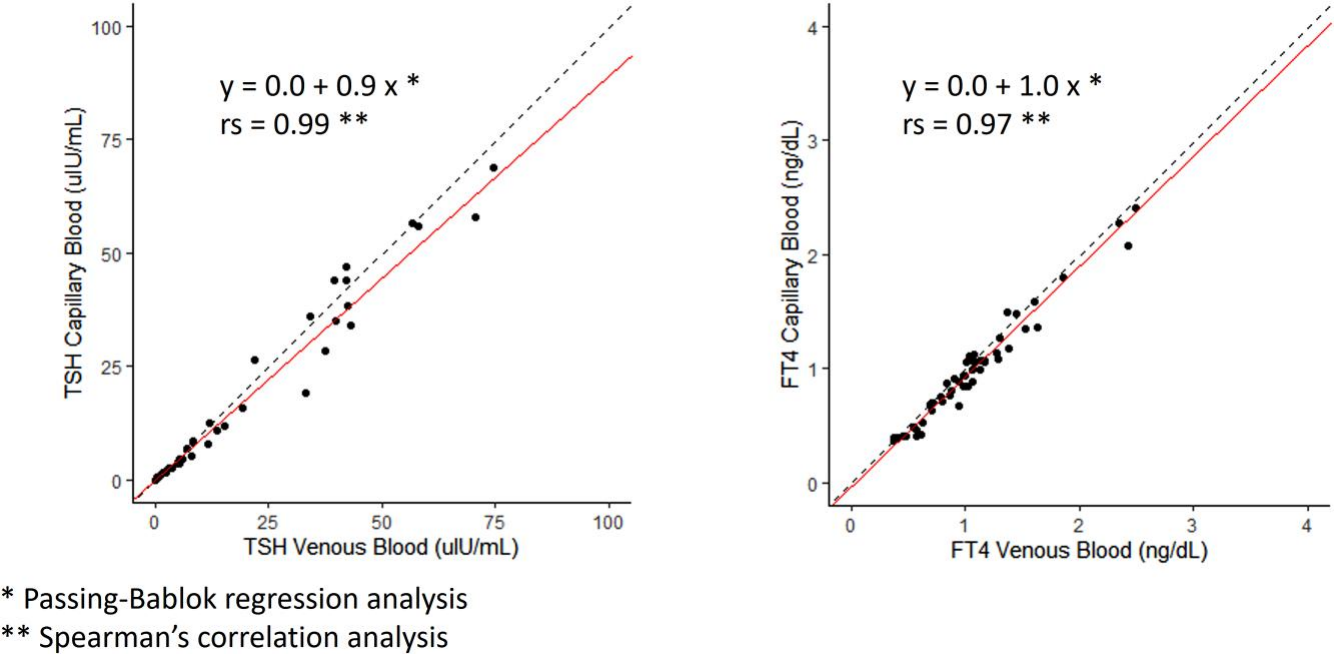
Correlations of TSH and FT4 levels between capillaries and venous blood in the FAS group. Linear scale scatter plots of capillary and venous blood TSH and FT4 with regression lines from Passing–Bablok regression analyses. TSH and FT4 levels in capillary and venous blood were strongly correlated. The straight line shows the Passing–Bablok regression line, and the dotted line shows the line with slope = 1 and y-intercept = 0. FAS, full-analysis set; FT4, free T4.

TSH and FT4 levels were successfully measured in 49 patients in the FAS group using capillary blood collected via TAP II. One patient was excluded because the value exceeded the measurable range (0.01-100 μIU/mL) of the d-IA used in this analysis. The primary objective of this study was to measure TSH and FT4 levels in capillary blood and compare them with venous blood values. The success rate of blood collection using TAP II was 89.1% (49/55). Capillary and venous blood TSH values were strongly correlated in the FAS group (rs = 0.99, *P* < .05; Spearman's correlation analysis) (Fig. 2), and the slope was y = 0.0 + 0.9 x (Passing–Bablok analysis). The PPS group also had a strong correlation between capillary and venous blood TSH values (rs = 0.99, *P* < .05), and the slope was y = 0.0 + 0.8 x.

In the analysis of the FT4 value, a strong correlation was observed between capillary and venous blood FT4 values in the FAS group (rs = 0.97, *P* < .05) (Fig. 2), and the slope was y = −0.0 + 1.0 x. Similar to the analysis in the FAS group, capillary and venous blood FT4 values were also strongly correlated in the PPS group (rs = 0.98, *P* < .05), and the slope was y = −0.0 + 1.0 x. However, 1 of the 24 participants was excluded from the analysis because the measurement exceeded the measurement range. Linear scale scatter plots of capillary vs venous blood with Passing–Bablok regression analyses for TSH and FT4 levels are shown in Fig. 2.

### Analysis of AST, ALT, T-eBil, and CRE Levels Between Capillary (TAP II) and Venous Blood

**Figure 3.**
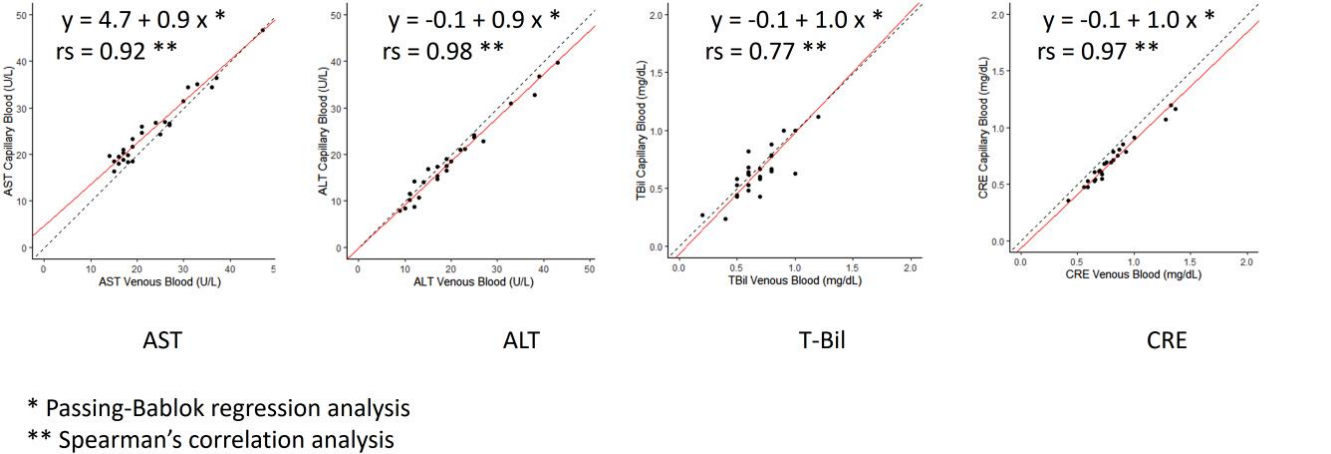
Correlations of AST, ALT, T-Bil, and CRE levels between capillaries and venous blood of 26 participants in the FAS group. Linear scale scatter plots of capillary and venous blood biochemical parameters with regression lines from Passing–Bablok regression analyses. Correlations were observed between capillary and venous blood in AST, ALT, T-Bil, and CRE levels. The straight line shows the Passing–Bablok regression line, and the dotted line shows the line with slope = 1 and y-intercept = 0. ALT, alanine aminotransferase; AST, aspartate aminotransferase; CRE, creatinine; FAS, full-analysis set; T-Bil, total bilirubin.

As a secondary analysis, biochemical parameters were successfully measured in 26 participants in the FAS group using capillary blood collected via TAP II, with a success rate of 47.3% (26/55). Correlations were noted between capillary AST, ALT, T-Bil, and CRE levels and venous blood levels (Fig. 3). The correlation coefficients were rs = 0.92 (*P* < .05), rs = 0.98 (*P* < .05), rs = 0.77 (*P* < .05), and rs = 0.97 (*P* < .05), respectively. All 4 parameters showed a significant correlation between capillary and venous blood, although the correlation of T-Bil was weaker than that of the other biochemical parameters.

### Analysis of TSH and FT4 Between d-IA and ARCHITECT Chemiluminescence kit

**Figure 4.**
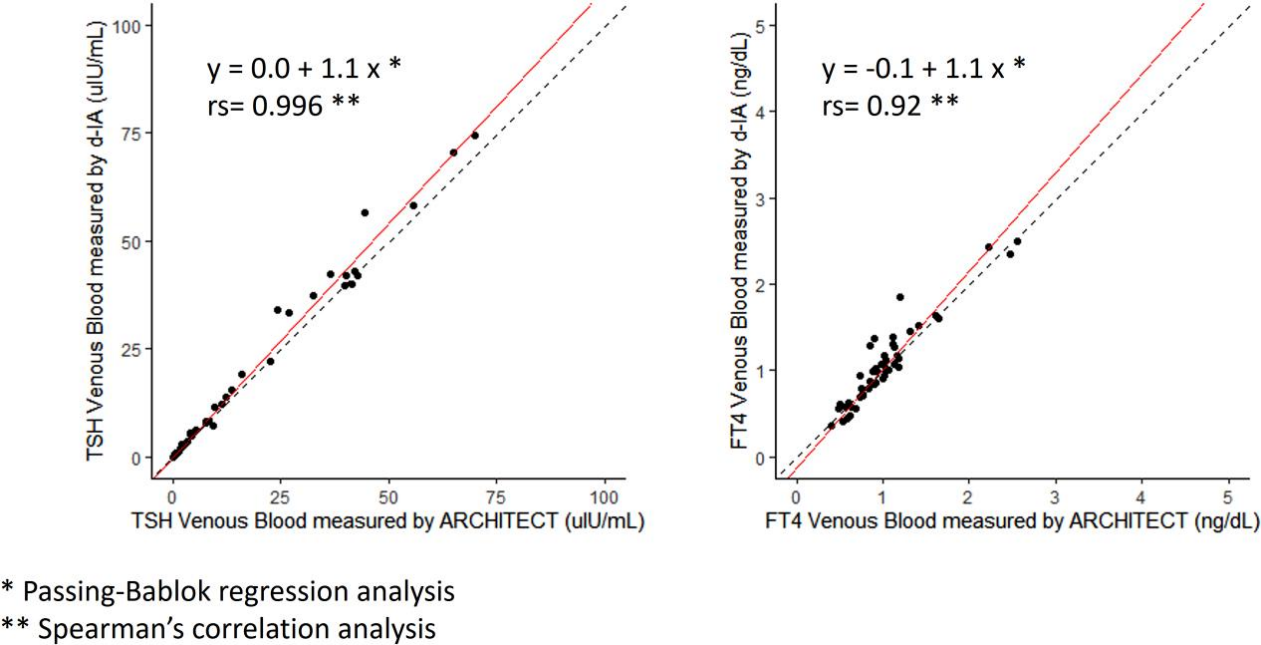
Correlations of venous TSH and FT4 levels between the digital immunoassay and ARCHITECT of 49 participants in the FAS group. Linear scale scatter plots of TSH and FT4 in venous blood measured with digital immunoassay and ARCHITECT with regression lines from Passing–Bablok regression analyses. TSH and FT4 levels were correlated between capillary blood and the ARCHITECT values. The straight line shows the Passing–Bablok regression line, and the dotted line shows the line with slope = 1 and y-intercept = 0. FAS, full analysis set; FT4, free T4.

To confirm the accuracy of the currently used d-IA data of venous blood, the TSH and FT4 values of venous blood d-IA levels were compared with those of the ARCHITECT chemiluminescence assay. The analyses were performed in 50 patients for FT4 evaluation and in 49 of 50 patients for TSH evaluation because 1 patient exceeded the measurable range. A strong correlation was observed between TSH and FT4 levels in the venous blood d-IA and ARCHITECT in the FAS group. The correlation coefficients were as follows: rs = 1.0 (*P* < .001) and rs = 0.92 (*P* < .001), respectively (Fig. 4). The same analyses were performed in the PPS group (24 patients), and a strong correlation was observed between the 2 measurement methods: rs = 1.0 (*P* < .001) and rs = 0.91 (*P* < .001), respectively.

## Discussion

Our results showed that the success rate of TAP II blood collection was 89% (49/55), and notable correlations were observed between capillary and venous blood for both TSH and FT4. According to the guidelines of the American Thyroid Association [23] and European Thyroid Association [24], serum TSH levels should be measured as an initial screening for thyroid dysfunction as it has the highest sensitivity and specificity. However, it is challenging to distinguish overt hyper/hypothyroidism from subclinical hyper/hypothyroidism or to detect central hypothyroidism by measuring only the TSH value. Woo et al developed a novel method to measure T4, T3, and TSH simultaneously from the same sample using a combination of multi-immunoreaction-based dual-color capillary electrophoresis and a dual-color laser-induced fluorescence technique [25]. They showed this method had high detection sensitivity and required a short measurement time. However, FT4 is the hormone that represents the actual thyroid hormone status better than T4, and, thus, FT4 is used in daily clinical practice to evaluate thyroid function combined with TSH values. Consistent with previous reports [20, 21], our study demonstrated that capillary TSH and FT4 levels strongly correlate with venous blood values, suggesting that capillary values may be used to assess actual thyroid function in adults.

The biochemical parameters were measured as secondary analyses using the same plasma obtained via TAPII and venipuncture. While using anti-thyroid drugs, adverse events such as liver injury [26] should be carefully monitored. The results showed a correlation between AST, ALT, T-Bil, and CRE, although the correlation coefficient was not strong for T-Bil (rs = 0.77). Wickremsinhe et al observed a nearly perfect concordance, with concordance correlation coefficients of > 0.99 [9] when comparing the biochemical parameter values of AST, ALT, and T-Bil between the capillary and venous blood. The difference in the concordance of T-Bil between our study and that of Wickremsinhe et al might be due to hemolysis. To detect hemolysis, they used both potassium levels in the blood and an automated mechanical system, and their results showed comparable quality to that of venipuncture blood. However, they also reported that the prevalence of hemolysis was higher in capillary blood than in venous blood. Because our study did not measure potassium levels, nor did we use an automated evaluation system, we could not evaluate hemolysis. As hemolysis affects each parameter differently, this difference in correlation may appear only for T-Bil. Considering the overall correlations in biochemical parameters, these values in capillary blood can also be used as true values for adults if we are cautious about the effect of hemolysis.

The TAP II device enabled us to collect more than 125μL of capillary blood in 26 participants, and we proved that this amount is enough to evaluate at least 6 parameters, including TSH and FT4. Several studies have successfully evaluated biochemical parameters and immune markers in capillary blood samples collected from the upper arm via a self-sampling device [8-11]. Furthermore, capillary samples were shipped overnight with or without centrifugation under controlled conditions to maintain sample quality in these studies. This suggests that utilizing a self-blood collection device in telemedicine may be feasible and reliable in several fields of internal medicine.

This study has some limitations. First, we did not evaluate some considerations relating to logistic aspects, such as the stability of the self-collected blood sample, costs for reagents and shipments, availability of the analyzers in the local hospitals, time to get the result back, way to communicate the results to the physicians of those patients, and costs of this new consultation. Second, as mentioned earlier, we did not evaluate hemolysis using potassium levels. Finally, our study did not reflect the participants’ perspectives. When this article was written, this device was not approved for commercial use in Japan. Thus, once approved, we should consider methods for collecting patient voices so that telemedicine using this device is more feasible, usable, and reliable for patients.

## Conclusion

TSH and FT4 levels in capillaries strongly correlate with venous blood values, and even trace samples can be used in high-precision d-IA methods. The results showed capillary blood can be measured using an autologous blood collection device without adverse events. These results may promote telemedicine in assessing thyroid function.

## Data Availability

\Restrictions apply to the availability of some or all data generated or analyzed during this study to preserve patient confidentiality or they were used under license.

## Clinical trial registration number

#CRB3180024, #IRB364
